# Supplementary material for: Are carbon emissions trading and green financial instruments synergistic? -Comprehensive quantitative research based on content analysis
Source: PLoS One. 2024 Mar 7;19(3):e0298601. doi: 10.1371/journal.pone.0298601 (PMC10919601; doi:10.1371/journal.pone.0298601)
Supplement: S1 Data — (ZIP) [file pone.0298601.s001.zip › Data/地级市Z_F工作报告.zip!/░▓╗╒/│╪╓▌╩╨/└┤╫╘╣1⁄2╓┌║┼í╛┬φ┐╦╩2╛▌═°í┐_2012.docx]

各位代表：
    现在，我代表市人民政府，向大会作工作报告，请予审议，并请市政协委员和其他列席人员提出意见。

一、2011年和本届政府工作回顾

    2011年，在省委、省政府和市委的坚强领导下，我们紧紧依靠全市人民，认真贯彻落实省第九次、市第三次党代会精神，坚持以科学发展为主题，以全面转型、加速崛起、兴市富民为主线，解放思想，抢抓机遇，主动作为，有效应对各种复杂形势，全面完成市二届人大六次、七次会议确定的目标任务。全市生产总值372.5亿元，增长13%；财政收入56.8亿元，增长30.9%；城镇居民人均可支配收入18925元，增长18.3%；农民人均纯收入6908元，增长18.5%。一年来，我们主要抓了以下六个方面工作：
    第一，坚持综合施策，经济保持平稳较快发展。工业经济发展加快。制定出台加快工业经济转型发展的意见和促进工业经济平稳运行的政策措施，大力增加工业投入，工业固定资产投资162.6亿元，增长41.2%，增幅位居全省第二，工业增长的内生动力进一步增强；引进光大银行在池设立分支机构，成立2家村镇银行、2家创业发展投资基金、1家典当公司、4家担保公司，18家小额贷款公司开业，九华股份上市力度加大，颐和新能源进入上市辅导期；全年新增贷款79亿元，较年初增长30%，增幅位居全省第一。规模以上工业增加值95亿元、增长22.9%，新增规模以上工业企业42户，工业化率37.5%、比上年提高3.1个百分点。农村经济稳定发展。兑现粮食直补、农机补贴等惠农政策资金1.7亿元；131家省市级农业产业化龙头企业实现销售收入58亿元、增长16%；天方集团成为全市首家国家级龙头企业，贵池梅里成为省级农业产业化示范区，东至东流、青阳九华进入第一批省级现代农业示范区行列；新增省级农民专业合作社示范社5家，省级标准化生产示范基地5个，省部级畜禽水产标准化规模养殖场13家，无公害农产品、绿色食品、有机农产品13个，农产品地理标志1个，地理证明商标1个；政策性农业保险承保面积181.4万亩；新建蔬菜基地2210亩。农业总产值94.2亿元，增长4.6%；粮食总产69.4万吨，增长2.1%。旅游等现代服务业不断壮大。制定出台加快发展旅游业实施意见和旅游市场营销奖励办法，全年接待国内外游客2075万人次、增长34.3%，实现旅游收入190亿元、增长37.6%，其中九华山接待游客490.1万人次、增长23%，创建4A级景区2家；“万村千乡”新建农家店38家，“新网工程”延伸网点158家，限额以上商贸流通企业新增39家；站前区专业市场营业额超过15亿元，江口物流基地三期、池州国际会展中心加快建设；成功举办2011中国•池州首届绿色运动大会和“三月三•池州杏花村诗会”，大型文化电视片《品读城市•池州》和原创动漫剧《九华小和尚》在央视首播，九华山庙会入选第三批国家级非物质文化遗产名录，大愿文化园主体工程即将竣工。社会消费品零售总额107.1亿元、增长17.6%，服务业增加值131.7亿元、增长9.6%。
    第二，明晰产业定位，工业转型升级步伐加快。首位产业培育取得突破。将电子信息产业确定为首位产业，引进龙头项目正威半导体产业园，以及磊鑫科技年产180万台平板电脑、派沃科技年产100万套空气能热水器等一批整机项目，产业链上下游配套的触摸屏、注塑件等项目成功入驻。池州高新区成为省新型工业化电子信息产业示范基地。新兴产业和传统产业发展加快。启动实施新兴产业培育计划、百户企业技改提升三年倍增计划，节能环保、生物医药、高端装备制造、新能源、材料等战略性新兴产业实现产值47.2亿元，增长30.6%；完成工业技改投资80.3亿元。自主创新能力提升。新增高新技术企业18家、创新型企业20家，筹建省级院士工作站1家、省级企业技术研发机构8家，60家企业与省内外高校院所签订合作协议73项；发明专利申请量和授权量分别增长1.4倍、6.5倍，增幅分别位居全省第二、第一；高新技术产业增加值23.5亿元，增长46.1%，增幅位居全省第一。
    第三，狠抓重大项目，经济增长后劲持续增强。全市固定资产投资289.8亿元，增长30.4%；列入省“861”行动计划项目198个，开工建设73个，竣工18个，完成投资170.7亿元，占年度计划的136.5%。重大工业项目进展顺利。投资亿元以上工业项目落实103个，铜冠有色年产10万吨锌、海螺三期后两条生产线、勤上光电一期、贵航特钢、西恩新材料一期、四通光电产业园1条生产线、华尔泰年产10万吨二氧化碳、泰合森一期、富莱茵汽车部件、替克斯阀门1条生产线等53个项目按期竣工或投产达效。重大交通基础设施项目加快推进。九华山机场完成工作量的70%，宁安城际铁路线下工程完成80%，望东长江公路大桥开工建设，东九高速公路池州段正在开展征地拆迁，池州长江公路大桥北岸选址已经确定。项目谋划工作取得突破。全市重点项目储备库首次突破1000个，其中200个重大项目完成规划设计达到可操作程度，列入省“861”计划的战略性新兴产业项目投资规模占总量的60%。开展未利用地整理，全年批准用地计划25281亩，争取“点供”用地重大项目3个。
    第四，深化示范区建设，对外开放水平不断提高。园区建设加快推进。全力服务江南产业集中区发展，市开发区升格为国家级经济技术开发区。全市园区新拉开框架面积25.3平方公里，新建标准化厂房100万平方米，园区规模工业实现增加值67.3亿元、占全市的74.7%。新开工10亿元以上大项目7个。招商引资成效明显。全年新引进各类项目306个，投产项目158个，到位资金257亿元，增长42.8%。三一重工机械、普洛康裕生物制药、华孚30万锭色纺、利达40万台柴油发动机等一批重大项目落地开工。对外开放不断扩大。全年进出口总额2.58亿美元，增长20.6%。海关实现一分钟通关，与南通海关建立转关通关合作机制。与瑞典斯文永阿市、韩国求礼郡等国际友城开展互访和经贸合作活动。
    第五，推进统筹发展，城乡环境面貌日益改善。城镇化率达46%。中心城市建设进展加快。编制完成城市总体规划（2009-2030）、东部新城核心区城市设计、城市森林公园总体规划。在全省率先开展低碳生态示范城市建设，全面启动国家森林城市创建工作，平天湖—丰收湖水系贯通工程开工建设，清溪河等三大水系贯通工程18公里水上观光游线正式开通。主城区雨、污水管网建设改造完成52.9公里，府儒学复建等项目建设进展顺利，清溪河堤顶道路维修等工程按期完工，秋浦河大桥、九华河大桥等18项市政重点工程完成投资10.3亿元。城乡基础设施不断完善。特色县区建设步伐加快，青阳县获得安徽省第二届文明县城称号，东至、石台县荣获安徽省文明县城创建工作先进县称号。完成水利投资5.5亿元，解决10.2万农村人口饮水安全问题，69座病险水库除险加固工程全面开工，7个中小河流治理工程加快建设，5个万亩圩口达标工程启动实施、加固堤长36.4公里。10个乡镇启动实施农村清洁工程。升级改造国省县乡公路200公里，实施农村公路危桥加固工程38座，完工9座。完成池青九客运班线公交化改造，开通大渡口—安庆跨江公交。139个村开展了为民服务“一网通”工程试点。生态建设持续强化。平天湖、秋浦河源国家湿地公园获批试点建设。成功创建国家级环境优美乡镇1个、国家级生态村3个，新增3个省级生态乡镇、5个省级生态村。人工造林7万亩。治理水土流失面积30平方公里。完成19个矿区矿产资源整顿整合，减少矿山开采点45个。节能减排完成省定目标。
    第六，保障改善民生，社会大局保持和谐稳定。33项民生工程和19件惠民实事共投入资金18.5亿元，增长85%，民生工程在全省考核中位居第四。就业和社会保障体系逐步完善。城镇新增就业2.04万人，城镇登记失业率3.9%。农民工技能培训和新型农民培训2.7万人。城乡居民养老保险试点工作全面启动实施，企业退休人员养老金待遇和失业保险金标准调整提高，“老工伤”人员工伤保险、未参保集体企业退休人员以及长期临时工养老保险等三类历史遗留问题得到妥善解决，城镇养老、医疗、失业、工伤、生育保险超额完成全年任务。农村五保户集中供养率达70%。开工建设保障性住房17735套，竣工8155套。开工建设安置房145.6万平方米，竣工101.5万平方米。改造农村危房4673户。社会管理不断强化。扎实开展“五级书记带头大走访”和“四民”专题活动，为民办实事8192件。深入推进“平安池州”建设，加强社会治安防控体系建设，严厉打击刑事犯罪活动，人民群众的安全感位居全省前列。矛盾纠纷和信访隐患排查化解工作扎实开展。产品质量和食品药品安全监管继续加强。安全生产形势保持平稳。物价监测调控成效显现，居民消费价格指数逐步回落。应急管理得到加强。和谐社区加快建设，基层基础不断夯实。社会事业全面发展。启动实施学前教育三年行动计划，贵池区被列入国家学前教育发展项目试点，中小学校舍安全工程加固改造42.1万平方米，新建留守儿童之家165所。基层医药卫生体制综合改革巩固完善，新型农村合作医疗住院实际补偿比提高11个百分点。建成25个乡镇综合文化站、236个广播电视“村村通”工程、228个农家书屋，全年送戏917场、送电影6000场。全国第三次文物普查全面完成。低生育水平保持稳定。“六五”普法全面启动。村务公开逐步完善。民族、宗教工作深入开展，妇女、儿童、老年人和残疾人权益得到保障，审计、统计、物价、人事、供销、气象、地震、对台、档案、保密、地方志、社会科学等各项工作得到加强，精神文明和民主法制建设深入推进，国防动员、人民防空和民兵预备役等工作取得新成效，争创全国双拥模范城“三连冠”通过验收。农村扶贫开发工作取得新成效，石台县获得省委、省政府批准比照执行大别山革命老区有关扶持政策。
    在推进经济社会发展的同时，我们不断加强政府自身建设，政府职能转变和管理创新工作成效明显。自觉接受人大的法律监督、工作监督和政协的民主监督，注意听取各民主党派、工商联、无党派人士和各人民团体的意见，办理人大代表议案1件、建议96件和政协委员提案242件。深入推进政务公开，建立健全网络政民互动平台，舆情应对处置机制逐步完善。加强反腐倡廉建设，与时俱进地健全和完善制度，强化重点领域、关键环节的行政监察和审计监督，坚决纠正损害群众利益的突出问题。
    回首本届政府任期工作，我们在市委的坚强领导下，以胡锦涛总书记两次视察安徽和温家宝总理视察池州重要讲话精神为指引，深入贯彻落实科学发展观，积极抢抓国家促进中部崛起、扩大内需、皖江城市带承接产业转移示范区建设等重大机遇，成功战胜历史罕见自然灾害的重大挑战，有效应对百年不遇金融危机的严重冲击，经受住了复杂多变宏观环境的严峻考验，经济社会取得了令人鼓舞的发展成就。本届政府任期的六年，是池州历史上经济发展最快的六年，城乡面貌变化最大的六年，社会事业投入最多的六年，人民群众受益最广的六年。2011年，地区生产总值是2005年的3.1倍，六年年均增长15%，高于全省平均水平1.6个百分点；财政收入是2005年的4.9倍，年均增长30.1%，高于全省平均水平4.1个百分点；全部工业增加值是2005年的4.6倍，年均增长23.6%，高于全省平均水平4个百分点；社会消费品零售总额是2005年的2.7倍，年均增长17.8%，高于全省平均水平1.7个百分点；城镇居民人均可支配收入是2005年的2.4倍，年均增长15.7%，高于全省平均水平1.7个百分点；农民人均纯收入是2005年的2.4倍，年均增长15.3%，与全省平均水平持平。成功创建“中国优秀旅游城市”、“国家园林城市”、“全国双拥模范城”。
    这些成绩的取得，是省委、省政府和市委坚强领导的结果，是市人大、市政协和社会各界鼎力支持的结果，是全市人民团结奋斗的结果。在此，我代表市人民政府，向全市人民，向广大工人、农民、知识分子、干部、驻池解放军指战员、武警消防官兵和公安政法干警，向各民主党派、各人民团体、社会各界人士和各位老领导、老同志，致以崇高的敬意!向关心、支持池州建设的中央和省驻池单位，向在我市创业的境内外投资者、建设者，向给予市政府高度信任和倾力支持的各位人大代表、政协委员，表示衷心的感谢!
    在看到成绩的同时，我们也清醒认识到，我市经济社会发展中也还存在不少困难和问题，主要表现在：工业经济不强、县域经济薄弱、经济总量不大仍是影响我市全面发展的关键问题，做强产业、调优结构、转型发展的任务仍然十分艰巨；人才力量与产业发展的迫切需求不相适应，土地、环境等要素约束加大，物价偏高压力仍存，部分中小微企业生产经营困难；社会管理和公共服务的能力与人民群众的愿望和要求还存在较大的差距；政府职能需要进一步转变，工作效率需要进一步提升，制度建设需要进一步加强，等等。对此，我们必须高度重视，用改革和发展的办法，在今后的工作中着力加以解决。

二、今后五年发展和2012年工作建议

    当前和今后一个时期，我们既面临着难得的历史机遇，也面临着严峻的挑战。中部崛起、扩大内需、承接产业转移示范区、主体功能区战略叠加实施，池州开发开放的机遇前所未有。同时，宏观环境错综复杂，影响经济发展的不稳定、不确定因素仍然较多，转型发展的任务艰巨繁重。我们要以高度的历史责任感，进一步解放思想，抢抓机遇，扎实苦干，奋力谱写幸福池州的新篇章。
    按照省第九次党代会和市第三次党代会精神，今后五年政府工作的指导思想是：坚持以邓小平理论和“三个代表”重要思想为指导，深入贯彻落实科学发展观，紧紧围绕建设国家级生态经济示范区和世界级旅游目的地，以科学发展为主题，以“全面转型、加速崛起、兴市富民”为主线，大力实施“生态立市、工业强市、旅游兴市、商贸活市、文化名市”发展战略，着力推进绿色发展、开放发展、创新发展、和谐发展，加快建设经济繁荣、环境优美、社会和谐、生活富裕的幸福池州，为全面建成小康社会奠定坚实基础。
    通过五年的努力，力争实现“六个翻番”，达到“一个赶上”、“一个同步”、“一个领先”，即：实现地区生产总值、固定资产投资、财政收入、城镇居民人均可支配收入、农民人均纯收入、服务业增加值“六个翻番”；人均主要指标赶上皖江城市带承接产业转移示范区的平均水平；城乡居民收入增长与经济发展速度保持同步；生态文明建设在全国领先。
    今后五年经济社会发展的重点任务：
    ——坚定不移加快经济发展。经济发展是建设幸福池州的物质基础。围绕加快做大经济总量、做强经济实力、转变经济发展方式、提升城市综合竞争力的目标，突出以工业化为核心，坚持有限目标，全力培育首位产业，抢占产业发展高位，加快特色园区建设，发展节能环保、生物医药、高端装备制造、新能源、材料等战略性新兴产业；以旅游业为突破口，推动现代服务业加快发展；以现代农业为基础，促进农业生产经营专业化、标准化、规模化和集约化。
    ——不失时机深化改革开放。改革开放是建设幸福池州的强大动力。把招商引资作为开放型经济的第一抓手，大力提升招商引资的质量和水平，在全社会营造浓厚的发展氛围。紧紧抓住皖江城市带承接产业转移示范区建设的历史机遇，加快发展，早见成效。积极参与泛长三角区域、皖南国际旅游文化示范区分工合作。扩大外经外贸。完善落实扶持非公有制经济政策，促进民营经济又好又快发展。倡导体制机制创新，深化重点领域和关键环节改革。
    ——统筹协调加强城乡建设。统筹协调是建设幸福池州的基本要求。着力增强中心城市带动能力，加大城市建设投入力度，推进特色县城建设，加快打造宜居宜业宜游的生态山水园林城市。加强以水利为重点的农村基础设施建设，实施农村环境综合整治项目，不断改善农村生产生活条件，积极创建优美乡村。加强重大基础设施建设，构筑便捷通畅的综合交通体系、稳定清洁的能源保障体系、现代高效的信息化服务体系。
    ——千方百计促进民生和谐。民生和谐是建设幸福池州的集中体现。大力发展实体经济和服务业，推动创业就业，提高城乡居民收入，平抑物价增长，着力提高人民群众的生活水平和质量。积极争取国家政策支持，增加有效投入，加快发展各项社会事业，推进基本公共服务均等化，让发展成果惠及全市人民。加强和创新社会管理，夯实基层基础工作，不断提升人民群众的安全感和满意度。
    今年政府工作的总体要求是：认真贯彻落实党的十七大、十七届六中全会以及中央、全省经济工作会议精神，紧紧围绕建设幸福池州目标，更加注重打造特色产业，更加注重扩大有效投入，更加注重统筹城乡发展，更加注重保障改善民生，稳中求进，好中求快，保持经济持续快速健康发展，促进社会和谐稳定。
    综合考虑各方面因素，今年经济社会发展的主要目标是：全市生产总值增长13%，财政收入增长18%，固定资产投资增长20%以上，规模以上工业增加值增长23%，城镇居民人均可支配收入增长13%，农民人均纯收入增长13%，城镇新增就业1.26万人，城镇登记失业率控制在4.5%以内，人口自然增长率控制在6‰以内，完成节能减排任务。
实现上述目标，重点抓好以下八个方面工作：
    （一）着力推进工业强市，加快做大经济总量
    突出工业强市发展战略，认真实施加快工业转型发展、促进工业经济平稳运行的政策措施，推进工业经济加快发展壮大。全力培育首位产业。市和池州经济开发区、高新区围绕电子信息首位产业，按照“首位产业、首位支持，政府引导、市场主导”的要求，集中力量、集中资源，推进正威半导体产业园一期项目加快建设，进一步引进上下游产业链项目，尽快形成电子信息产业集聚集群发展；围绕贵池前江工业园的材料产业、东至县的生物医药化工产业、青阳县的机械加工及装备制造产业、石台县的旅游生态产业，加快县区主导产业的培育。强化企业自主创新。制定完善支持企业自主创新、高端人才引进等激励政策，启动池州高新区科技孵化器建设，鼓励高层次人才、创新团队领办创办高新技术企业，力争新增10家省级企业技术研发机构、10家创新型（试点）企业、10家高新技术企业，专利授权量600件以上，争创5件安徽省著名商标、3个安徽名牌产品。全年高新技术产业增加值增长30%。促进工业稳定增长。加强工业经济运行监测调度，认真实施扶持中小微企业发展的政策措施，确保中小微工业企业贷款增量高于上年水平，加大地产品促销力度，扩大市内产品市场占有率。重点跟踪调度110个亿元以上项目，确保铜冠电子铜箔一期、磊鑫平板电脑、贵航特钢二期、利达40万台柴油发动机等重大项目按期投产，加快派沃空气能热水器、普洛康裕生物制药、超威新能源等项目建设，新增规模以上工业企业60户。加大传统产业技改升级力度，完成技术改造投资100亿元。大力发展战略性新兴产业，力争在节能环保、新材料、生物医药等方面取得突破。积极谋划产业项目，争取新增项目储备规模300亿元以上，确保列入省“861”项目库和省战略性新兴产业项目库份额超过全省平均水平。全年完成工业固定资产投资200亿元，增长28%以上。加强节能减排和环境保护。抓好重点行业、重点领域节能技术改造和工程建设，继续推进污水管网建设和污水处理厂规范达标运营，积极发展循环经济，加快国家可再生能源建筑应用示范城市、低碳生态示范城市建设，创建省级循环经济示范单位5家以上。强化资源集约节约利用，认真落实土地使用税征收和财政对节约集约用地的奖补政策，推进矿产资源开发整顿规范工作，有序开发和合理利用岸线资源。强化水资源管理工作。
    （二）着力壮大旅游经济，突出发展现代服务业
建立服务业统计、考核体系，全年服务业增加值增长10%以上。大力发展旅游特色经济。确保接待国内外游客2600万人次、增长30%以上，旅游总收入260亿元、增长30%以上。突出九华山龙头带动，以大愿文化园建成开放为契机，推动山上与山下旅游互动发展，推进九华山世界地质公园申报工作。突出主城区在全市旅游发展中的中心地位，采取政府规划引导、市场引进知名旅游企业运作的方式，加大旅游景点的开发力度，加快杏花村建设，在主城区打造一流景区；推进旅游产品集成与后包装的品牌研发，建成一批高星级宾馆、快捷式酒店、经济型酒店、旅游停车场，完成池州旅游口岸项目前期工作，力争齐山—平天湖成功申报国家级风景名胜区。突出市、山、县旅游联动发展，加快牯牛降、升金湖、九子岩等主要景区建设，升级改造景区旅游公路，发展乡村旅游、生态旅游等参与式、体验式旅游业态；建立市域范围内无障碍旅游机制，认真实施旅游市场营销奖励政策，充分发挥旅行社、游轮公司和旅游直通车运营公司等市场中介作用，尽快形成全市旅游串点连线成面的良好局面。加快发展金融业。确保全年新增信贷投放80亿元。完善地方金融服务体系，加大域外金融机构的引进力度，推进村镇银行的筹建和运营，发展担保、小额贷款、典当等业态，严格防范金融风险。扩大直接融资，确保2支以上创业投资引导基金挂牌运作，大力引进私募基金，推进九华股份和颐和新能源的上市进程，力争本届政府任期内实现3-5家企业上市。进一步整合提升融资平台，积极推广助保金、采矿权抵押、仓单质押、商标权使用权及旅游经营权质押、林权及水面经营权担保抵押等融资创新产品。繁荣发展商贸物流业。继续实施“万村千乡”和农超对接等工程，积极发展连锁经营、物流配送和电子商务等现代流通方式，推进专业市场提升区域辐射力，全市新增限额以上商贸流通企业40户。推动站前区商贸物流园区与江口港区物流基地联动发展，加大站前区对外招商力度，争创省级现代物流服务业集聚区。加快池州国际会展中心建设。规划建设站前区总部经济园。力争引进1-2家知名第三方物流企业。
    （三）着力发展现代农业，夯实农业发展基础
坚持以工业化提升农业、以城镇化带动农村、以一体化创新机制，推动“三化同步”。加快推进农业产业化。深入实施农业产业化“563”提升行动，制定转型倍增计划，全市新增省市级龙头企业20家、市级农民专业合作社示范社20个。大力实施水稻产业提升行动、粮食丰产科技工程、油菜棉花高产创建活动和畜牧业升级计划、水产跨越工程，确保粮食总产稳定在68万吨以上。完善蔬菜基地的产、供、销体系，着力解决蔬菜生产、运输和销售过程中的关键问题，让市民吃上“平价菜”、“放心菜”。力争新增1个省级现代农业示范区、15个市级以上畜禽水产标准化规模养殖示范场以及15个无公害农产品、绿色食品、有机食品认证。加强农资市场综合整治，确保农产品质量安全。加强水利等基础设施建设。全面实施水利八大工程，完成除险加固重点小型水库48座，加快5个万亩圩堤达标续建工程建设，开工建设清溪河、九华河等5个中小河流治理工程，推进双溪水库项目规划论证等前期工作，扩挖当家塘1000口，疏浚灌溉除涝沟渠100公里，加固堰坝100处，更新改造小型泵站3000千瓦，完成水利投资5亿元。完成县乡公路升级改造100公里，新开工农村公路危桥改造项目40个，完善农村公路养护机制。加快实施新一轮农村电网改造升级工程。加快新农村建设。加大涉农资金整合力度，分块连线成片统筹实施优美乡村创建、农村清洁工程、农村危房改造和村庄整治工程，提升新农村示范点建设水平。进一步完善农村土地流转服务体系，推进土地承包经营权流转健康发展。加强耕地和基本农田保护，加大城乡建设用地增减挂钩和农村土地整治力度。加强农业技术推广、动植物疫病防控、农产品监管等公共服务机构建设，构建新型农业社会化服务体系。健全村级公益事业“一事一议”财政奖补工作长效机制。继续推进集体林权制度配套改革。继续落实强农惠农富农政策，扩大政策性农业保险覆盖面。
    （四）着力抓好基础建设，促进城乡统筹发展
    以加快城乡基础设施建设为重点，推进城乡、区域协调联动发展，着力提升城镇化水平，确保城镇化率达到47%。提升中心城市品位。按照“强化功能、提升品质、体现生态、宜居宜游”的要求，围绕生态山水园林城市特色，完成新一轮城市总规报批和城市建筑风貌等专项规划、控制性详规，强化规划的引领作用。提升城市功能，市本级实施政府投资项目68个，今年计划投资28.1亿元，启动主城区城市森林公园、平天湖国家湿地公园建设，加快平天湖—丰收湖水系贯通、污水管网等工程建设进度。加强房地产市场调控，促进房地产业持续健康稳定发展。加强城市管理，健全小区物业管理长效机制，推进市政园林公用设施分类精细化管理，加快公交车辆线路延伸和城市公交一体化进程，加大市容市貌、征收拆违、交通秩序、超限超载超速的管理与治理力度。推进交通基础设施建设。确保九华山机场建成通航，力争宁安城际铁路池州段建成，加快望东长江公路大桥、东至—九江高速公路、秋浦河大桥、九华河大桥、迎宾大道、牛头山港区公用码头建设，坚定不移地推动池州长江公路大桥、南沿江快速通道、殷汇—石台—祁门高速、池州—九江城际铁路、池州—九华山—黄山城际铁路、阜阳—池州—景德镇铁路池州段、江口港区铁路专用线等重大基础设施项目。完成池青九快速通道延伸至青阳工程，启动尧渡—东流一级公路前期工作，力争吴田—红石公路列入省道拓宽改造，升级改造国省道路网80公里。促进区域协调发展。制定分类指导区域经济加快发展的考核激励措施，推进各县区发挥自身优势，大力发展特色经济。加快各县老城改造和新区整体联动发展步伐，完善提升公共服务和居住功能，着力打造各具特色的中小城市。按照小城市标准培育发展10个特色镇。深入推进生态建设。以创建国家森林城市为抓手，大力实施城市园林、生态公益林、长江防护林、自然保护区等生态保护工程，启动主城区清溪河等水环境整治，着力构筑“山水环绕、城乡一体、碧水蓝天”的城乡生态建设格局。
    （五）着力强化招商引资，推进承接产业转移
    大力开展招商引资和开放合作，全面提高示范区承接水平。加大招商引资力度。突出特色产业招商，全面摸排上下游配套企业和关联产业，充分运用和拓展延伸央企、知名民企、长三角、珠三角、港澳台“五大合作”平台，制定“产业招商线路图”，主攻引进一批核心龙头型大项目和集群配套型项目。突出领导带队招商，按月调度通报签约项目跟踪落实情况。突出以商引商，完善招商政策体系并及时兑现，注重发挥各类商会、行业协会、中介组织和企业、园区招商主体作用。突出优化环境招商，把招商引资作为全社会的共同责任，严格招商引资考核奖惩，进一步营造重视招商、主动招商、支持招商、服务招商的浓厚氛围。确保全年引进规模项目300个，到位资金300亿元、增长20%以上。夯实产业承接平台。全力推进江南产业集中区建设，加快市区路网、供水、供电、供气等基础设施与集中区规划和建设的衔接，高起点规划、大手笔建设集中区。推进市开发区进一步完善功能，提高辐射力和带动力。提升5个省级开发区的承载能力。努力扩大外经外贸。加快有色金属、机械装备制造、特色农产品、生物医药化工、节能环保等进出口产业基地建设，力争池州港二期列入国家“十二五”口岸发展规划并通过国家验收，进一步优化口岸通关服务。全年外贸进出口总额增长15%，实际利用外资增长10%。
    （六）着力加强文化建设，推动文化发展繁荣
    以发展文化产业为重点，加快完善公共文化服务体系，推进文化大发展大繁荣。加快发展文化产业。实施重大文化产业项目带动战略，推进大愿文化园创建省级文化产业示范基地，加快动漫产业基地的规划建设，大力开展文化产业项目的招商，力争文化产业项目库新增100个，引进5个千万元以上文化产业项目。以国家和省级非物质文化遗产为依托，整合地方特色文化资源，运用市场机制培育以游客为目标群体的演艺市场，推进文化与旅游的深度融合。文化产业增加值增长25%以上。繁荣发展文化事业。深入实施文化惠民工程，建成使用市广播电视新中心，建成开放市博物馆、图书馆、文化馆、科技馆、青少年活动中心，启动市综合档案馆规划等前期工作。加强文物、非物质文化遗产保护、管理和利用。完善文化产品评价体系和激励机制，推出一批具有池州特色、符合时代特征的精品力作。完成《池州市志》编纂工作。切实增强文化凝聚力。坚持以社会主义核心价值体系引领社会思潮，进一步加强社会公德、职业道德、家庭美德和个人品德教育，加强政务诚信、社会诚信和司法公信建设，加强文明城市、文明社区、文明村镇、文明行业、文明单位、文明景区等创建工作，形成良好的社会风尚和公共秩序。
    （七）着力保障改善民生，不断增进人民福祉
    坚持把保障和改善民生作为一切工作的出发点和落脚点，不断提高人民群众生活水平。认真实施居民收入倍增规划。把促进就业创业作为增加城乡居民收入的主渠道，在全社会掀起创业造富、就业增富、勤劳致富的热潮，推动愿拼搏的人创业、有技能的人就业、肯干事的人增收，确保城乡居民收入增幅高于全省平均水平。扎实推进新一轮扶贫开发，确保贫困地区农民人均纯收入增幅高于全市平均水平。拓展提升民生工程。按照尽力而为、量力而行、滚动发展的原则，认真实施省33项民生工程，建立健全稳定多元的筹资机制，加强项目管理和考核奖惩，不断提高民生工程的质量效益。继续办好12件政府惠民实事，让发展成果惠及全市人民。抓好就业和社会保障工作。多渠道开发就业岗位，做好重点群体的就业工作，加强对被征地农民的培训，提高被征地农民就业能力。深入实施创业富民工程，打造一批创业示范园区，不断提升园区企业入驻率和吸纳就业率，开展新型农民培训1.35万人、劳动者就业技能培训0.9万人，促进劳动力就近就地就业。全面实行医疗、生育保险市级统筹，完善统筹城乡的养老保险制度。加强社会保险扩面征缴。完善临时救助制度，提高社会救助水平。开工建设保障性住房11019套，竣工13964套。推进各项社会事业全面进步。优先发展教育事业，深入实施学前教育三年行动计划，推进义务教育均衡发展，全面完成中小学校舍安全工程，确保70%以上城乡义务教育学校实现标准化，大力发展职业教育，推动高等教育和特殊教育健康发展，加强学生交通安全管理。大力发展卫生事业，巩固扩大基层医药卫生体制综合改革成果，稳步推进县级公立医院综合改革试点，进一步完善医疗卫生服务体系，加强血防工程建设，抓好重大传染病和职业病防控。继续实施全民健身计划。继续加强人口计生工作，控制出生人口性别比，提高出生人口素质。加强统计基层基础建设。充分发挥工会、共青团、妇联等人民团体的重要作用，加强外事、侨务、对台、供销、气象、保密、档案、地方志、防震减灾、社科研究等各项工作。重视养老服务、红十字和福利慈善事业，推动养老产业发展。
    （八）着力创新社会管理，加强和谐池州建设
    坚持依法治市，着力夯实基层基础，不断提高社会管理科学化水平。加强基层社会管理体系建设。推动社会建设和管理重心下移，提升基层社会管理能力，配足配强社区干部队伍，推进社区服务用房和信息化建设，强化城乡社区自治和服务功能。深化村务公开民主管理，推进为民服务“一网通”工作，促进政府行政管理与基层群众自治有效衔接、良性互动。加强和改进新形势下群众工作。深化各级干部大走访活动成果，认真落实联系群众“一会三制”和信访工作责任制，健全完善领导干部接访、社会矛盾排查化解、社会舆情分析研判、社会稳定风险评估长效机制，引导群众依法维护自身合法权益，切实解决群众反映的合理诉求和热点、难点问题，把各种不稳定因素化解在基层、消除在萌芽状态，着力解决影响社会和谐稳定的源头性、基础性、根本性问题。深入推进平安池州建设。加强社会治安防控体系建设，扎实开展“六五”普法工作，依法防范和打击违法犯罪活动。严格落实安全生产责任制，加大重点行业和领域隐患排查治理以及安全专项整治力度，严防重特大事故发生。加强食品药品安全监管，严厉打击食品药品安全违法行为。加强自然灾害防治工作，提高防灾减灾能力。加强和改进价格调控，抓好涉及民生的价格监管工作。完善应急管理体制机制和预案体系，提高保障公共安全和处置突发事件的能力。依法管理民族宗教事务，保持民族宗教领域和谐稳定。积极支持驻池部队建设，加强国防教育，做好国防动员、人民防空和民兵预备役工作，巩固发展双拥模范城创建成果。

三、全面加强政府自身建设

    实现新一届政府的奋斗目标，完成今年各项工作任务，必须全面加强政府自身建设，努力提升科学理政、依法行政、高效施政、廉洁从政的能力和水平。
    （一）抓学习，强化发展意识。深入推进学习型政府建设，进一步解放思想，认真学习研究宏观政策，顺应宏观形势变化，把握经济发展规律，敢于突破一切不符合科学发展的思维定式，不断提升工作创新能力。
    （二）抓宗旨，强化群众观念。坚持以人为本、执政为民，牢固树立群众观念，进一步增强宗旨意识，创新联系群众方式，想事情、看问题、抓工作都能从大局和群众利益出发，立足长远，努力解决事关民生的根本性问题。
    （三）抓法治，强化依法行政。深入推进法治政府建设，严格依照法定权限和程序行使职权，完善重大行政决策听证、合法性审查、事后评价制度。主动接受人大及其常委会的依法监督和政协民主监督，切实办好人大代表议案建议和政协提案工作。充分听取民主党派、工商联、无党派人士和各人民团体意见建议，自觉接受群众监督、新闻媒体和网络监督。
    （四）抓作风，强化工作执行。进一步转变政府职能，深入推进电子政务、政务公开和政府信息公开，加强政务服务体系建设，启动实施事业单位分类改革，深化行政审批制度改革，优化审批流程，提升行政效能。加强政务督查、效能监察和绩效评价，深入开展市直机关单位作风评议等活动，坚决查处影响投资环境的人和事。严格落实目标管理责任制、重点工作调度制、重大事项督办制，做到责任分解到人、措施落实到事、目标实现到位。
    （五）抓廉政，强化制度保障。严格落实党风廉政建设责任制，不断提高拒腐防变能力。深入推进节约型机关建设，严控“三公”经费。加强领导干部经济责任以及政府性投资项目审计，进一步强化财政预算、政府采购、国有土地出让、岸线和矿产资源开发、国有资产转让、招投标交易、人事编制管理，坚持用制度管钱、管事、管资源。坚决制止和纠正损害群众利益的不正之风。加强对政府班子自身和公务员的教育、管理和监督，以敬畏心对待权力，以公德心对待社会，以责任心对待工作，努力建设一支政治坚定、业务精湛、作风过硬、纪律严明的公务员队伍。

    各位代表！前景无限美好，责任催人奋进。让我们更加紧密团结在以胡锦涛同志为总书记的党中央周围，在省委、省政府和市委的坚强领导下，把人民的期盼化为前进的动力，把发展的使命变成积极的行动，解放思想，开拓奋进，加快建设经济繁荣、环境优美、社会和谐、生活富裕的幸福池州，以优异成绩迎接党的十八大胜利召开！
